# Conceptual and operational definition of nursing outcomes regarding the breastfeeding establishment*

**DOI:** 10.1590/1518-8345.3007.3259

**Published:** 2020-04-17

**Authors:** Suellen Cristina Dias Emidio, Flávia de Souza Barbosa Dias, Sue Moorhead, Jennifer Deberg, Ana Railka de Souza Oliveira-Kumakura, Elenice Valentim Carmona

**Affiliations:** 1 Universidade Estadual de Campinas, Faculdade de Enfermagem, Campinas, SP, Brazil.; 2 Scholarship holder at the Coordenação de Aperfeiçoamento de Pessoal de Nível Superior (CAPES), Grant # 38P-4842/2018, Brazil.; 3 University of Iowa, College of Nursing, Iowa, IA, United States of America.; 4 University of Iowa, Hardin Library for the Health Sciences, Iowa, IA, United States of America.

**Keywords:** Breast Feeding, Nursing, Nursing Process, Validation Studies, Outcome Assessment, Review, Aleitamento Materno, Enfermagem, Processos de Enfermagem, Estudos de Validação, Avaliação de Resultados, Revisão, Lactancia Materna, Enfermería, Proceso de Enfermería, Estudios de Validación, Evaluación de Resultado, Revisión

## Abstract

**Objective:**

to construct conceptual and operational definitions of Nursing Outcomes “Breastfeeding establishment: infant (1000)” and “Breastfeeding establishment: maternal (1001)”.

**Method:**

integrative literature review in the following databases: PUBMED (United States National Library of Medicine); LILACS (Latin American and Caribbean Health Sciences Literature); CINAHL (Cumulative Index to Nursing and Allied Health Literature); SciVerse SCOPUS; Web of Science; BDENF (Brazilian Nursing Database) and EMBASE (Excerpta Medica Database). The gray literature was explored to elucidate topics not covered by the articles. Of 3242 articles, 96 were selected to be read in full, and 43 were used for constructing the definitions. Five theses, three dissertations, three books and two manuals were selected.

**Results:**

all the results were reviewed. The definitions facilitated the improvement of the content proposed by the Nursing Outcomes Classification, favoring its application in clinical practice and supporting the development of research and teaching.

**Conclusion:**

it was proposed to change the definition of the two outcomes, as well as to change the title of one of them to “Breastfeeding establishment: newborn & infant” (1000), modifying seven of its indicators and excluding one. For the outcome related to the mother, it was proposed to modify two indicators and exclude one.

## Abstract (pt)

**Objetivo:**

construir definições conceituais e operacionais dos Resultados de Enfermagem “Estabelecimento da amamentação: lactente (1000)” e “Estabelecimento da amamentação: mãe (1001)”.

**Método:**

revisão integrativa da literatura nas bases de dados: PubMed (Biblioteca Nacional de Medicina dos Estados Unidos); LILACS (Literatura Latino-Americana e do Caribe em Ciências da Saúde); CINAHL (Índice Cumulativo de Enfermagem e Literatura Aliada em Saúde); *SciVerse* SCOPUS; *Web of Science*; BDENF (Base de Dados de Enfermagem Brasileira) e EMBASE (*Excerpta Medica Database*). A literatura cinzenta foi explorada para a elucidação de tópicos não abordados pelos artigos. De 3242 artigos, 96 foram selecionados para a leitura na íntegra e 43, utilizados na construção das definições. Selecionaram-se cinco teses, três dissertações, três livros e dois manuais.

**Resultados:**

todo o conteúdo dos resultados foi revisado. As definições facilitaram o aprimoramento do conteúdo proposto pela *Nursing Outcomes Classification*, favorecendo sua aplicação na prática clínica e sustentação para o desenvolvimento de pesquisas e ensino.

**Conclusão:**

foi proposta mudança na definição dos dois resultados, bem como alteração do título de um deles para *Estabelecimento da amamentação: recém-nascido & lactente* (1000), com alterações em sete de seus indicadores e a exclusão de um. Para o resultado relacionado à mãe, foram propostas modificações em dois indicadores e exclusão de um.

## Abstract (es)

**Objetivo:**

construir definiciones conceptuales y operacionales de los Resultados de Enfermería “Establecimiento de la lactancia materna: lactante (1000)” y “Establecimiento de la lactancia materna: madre (1001)”.

**Método:**

revisión integrativa de la literatura en las bases de datos: PUBMED (Biblioteca Nacional de Medicina de los Estados Unidos); LILACS (Literatura Latinoamericana y del Caribe en Ciencias de la Salud); CINAHL (Índice Acumulativo de Enfermería y Literatura Aliada en Salud); SciVerse SCOPUS; Web of Science; BDENF (Base de Datos de Enfermería Brasileña) y EMBASE (Excerpta Medica Database). Se exploró literatura gris para dilucidar tópicos no abordados por los artículos. De 3242 artículos, se seleccionaron 96 para lectura completa y se emplearon 43 en la construcción de las definiciones. Se seleccionaron cinco tesis, tres disertaciones, tres libros y dos manuales.

**Resultados:**

se revisó todo el contenido de los resultados. Las definiciones facilitaron el perfeccionamiento del contenido propuesto por *Nursing Outcomes Classification*, lo que favorece su aplicación en la práctica clínica y respaldo para desarrollo de investigaciones y enseñanza.

**Conclusión:**

se propuso un cambio en la definición de los dos resultados, así como la modificación del título de uno de ellos a Establecimiento de la lactancia materna *: recién nacido y lactante* (1000), con modificaciones en siete de sus indicadores y la exclusión de un indicador. Para el resultado relacionado con la madre, se propusieron modificaciones en dos indicadores y la exclusión de un indicador.

## Introduction

The World Health Organization (WHO) recommends that breastfeeding is offered exclusively until the sixth month and complemented until the child is two years of age or older, because it favors growth and development^(^ 1 ^)^. In addition, breastfeeding women are at decreased risk of postpartum hemorrhage, breast cancer, cardiovascular disease, and type 2 Diabetes mellitus^(^ 2 ^)^.

Brazil follows these WHO recommendations and has an extensive legal framework protecting the rights of women and children in this context, providing the necessary conditions for the establishment and maintenance of lactation^(^ 3 ^)^. However, early weaning is still part of the Brazilian reality, and the prevalence of Exclusive Breastfeeding (EBF) in children under six months old is only 36.6%^(^ 4 ^)^.

The literature points out that the first weeks of breastfeeding are crucial for the maintenance of lactation and reduction of early weaning. Some difficulties occur within the first 24 hours, such as the baby’s difficulty latching on and sucking, nipple pain and injury, breast engorgement, perception of insufficient milk supply, and maternal fatigue. These aspects may reflect the woman’s satisfaction with breastfeeding, as well as the actual supply of human milk, increasing the chance of introducing other foods, which could lead to weaning^(^ 5 ^-^ 8 ^)^.

In their clinical practice, nurses should identify the needs of mothers and babies in the process of Breastfeeding establishment. Continuous assessment of the pair, considering relevant indicators for the success of EBF, may be based on the application of the Nursing Outcomes (NO) and the Nursing Outcomes Classification (NOC). The NO “describe a state, behavior, or perception of the individual, family, or community, measured over a *continuum* in response to a nursing intervention or interventions”^(^ 9 ^)^.

For the indicators proposed by the NOC to be useful in clinical practice, validation studies enhancing their content must be developed. These studies need to be performed with different populations to allow the generalization of the NO and their use by different professionals in different clinical settings^(^ 9 ^-^ 10 ^)^.

The literature review is the first stage of validation studies involving nursing classifications, being fundamental for the development of conceptual and operational definitions^(^ 11 ^-^ 13 ^)^. Conceptual definitions are the articulated abstraction of a phenomenon, in order to facilitate the understanding of the research variables. Operational definitions, on the other hand, result from a procedure that assigns communicable meaning to a concept, i.e., an accurate description of how to evaluate the phenomenon in question^(^ 14 ^-^ 16 ^)^. Thus, performing a literature review to construct these definitions can help nurses identify the phenomenon of interest and evaluate the indicators in a more accurate and standardized manner.

Given the above, the objective of this study was to construct the conceptual and operational definitions of NO “Breastfeeding establishment: infant (1000)” and “Breastfeeding establishment: maternal (1001)”, proposed by the NOC^(^ 9 ^)^. The construction of these definitions is the first step of a clinical validation study focused on these two NO.

## Method

This integrative review (IR) was conducted according to the methodological approach developed by Whittemore and Knafl^(^ 17 ^)^, following the recommendations of the Preferred Reporting Items for Systematic Reviews and Meta-Analyses - PRISMA^(^ 18 ^)^. The questions that guided the review were: “How is breastfeeding established?” and “What are the signs and characteristics of the Breastfeeding establishment, considering babies and/or mothers?”.

The bibliographic search was carried out from August to September 2017, and the electronic databases used were: PUBMED (United States National Library of Medicine); LILACS (Latin American and Caribbean Health Sciences Literature); CINAHL (Cumulative Index to Nursing and Allied Health Literature); SCOPUS; Web of Science; BDENF (Brazilian Nursing Database) and EMBASE (Excerpta Medica Database).

The inclusion criteria were: original, complete studies, in Portuguese, English or Spanish, published from 2013 to 2017 and that addressed the Breastfeeding establishment for babies and mothers, or the challenges and failures thereof. We opted for limiting the study period to the last five years because important changes in concepts related to breastfeeding occurred during this time. Letters, editorials, case studies, pilot studies and literature reviews were excluded.

The studies were located using Medical Subject Headings Terms and Health Sciences Descriptors. Furthermore, the keywords were chosen based on the title of the NO studied, by combining the terms “breastfeeding” or “weaning” with “infant” or “newborn” or “mother” and “establishment” or “success” or “failure” or “obstacle” or “barrier” or “challenge”. These terms were searched independently at first, and then in combination, with the help of a librarian (Table 1).

**Table 1:** Descriptors and terms used in the selection of studies for the integrative review. Campinas, SP, Brazil, 2017

Database	Descriptors/ Keywords	Total articles found*	Total after removing duplicates	Total after reading the titles and abstracts	Selected to be read in full	Selected to be included
**Lilacs^†^**	Newborn; Infant; Breastfeeding; Weaning	353	74	46	08	2
**Bdenf^‡^**	Newborn; Infant; Breastfeeding; Weaning	353	124	149	06	2
**Cinahl^§^**	Infant, Newborn; Breastfeeding; Weaning/Time Factors; Success; Establishment; Challenge; Failure; Obstacle; Barrier	632	156	117	23	14
**Pubmed^||^**	Infant, Newborn; Breastfeeding; Weaning	575	154	108	12	3
**Embase^¶^**	Infant, Newborn; Breastfeeding; Weaning/MotherTime Factors; Success; Establishment; Challenge; Failure; Obstacle; Barrier	363	135	141	9	0
Scopus**	Infant, Newborn; Neonate; Breastfeeding; Weaning/ Mother; Time; Duration; Success; Establishment; Challenge; Failure; Obstacle; Barrier	473	233	148	23	13
**Web of Science**	Infant, Newborn; Neonate; Breastfeeding; Weaning/ Mother; Time; Duration; Success; Establishment; Challenge; Failure; Obstacle; Barrier	493	169	136	15	9

* Articles selected with limitations: time (2013 to 2017) and language;

† Lilacs = Latin American and Caribbean Health Sciences Literature;

‡ Bdenf = Brazilian Nursing Database;

§ Cinahl = Cumulative Index to Nursing and Allied Health Literature;

|| PubMed = United States National Library of Medicine;

¶ Embase = Excerpta Medica Database;

** Scopus = SciVerse Scopus

**Figure 1:**
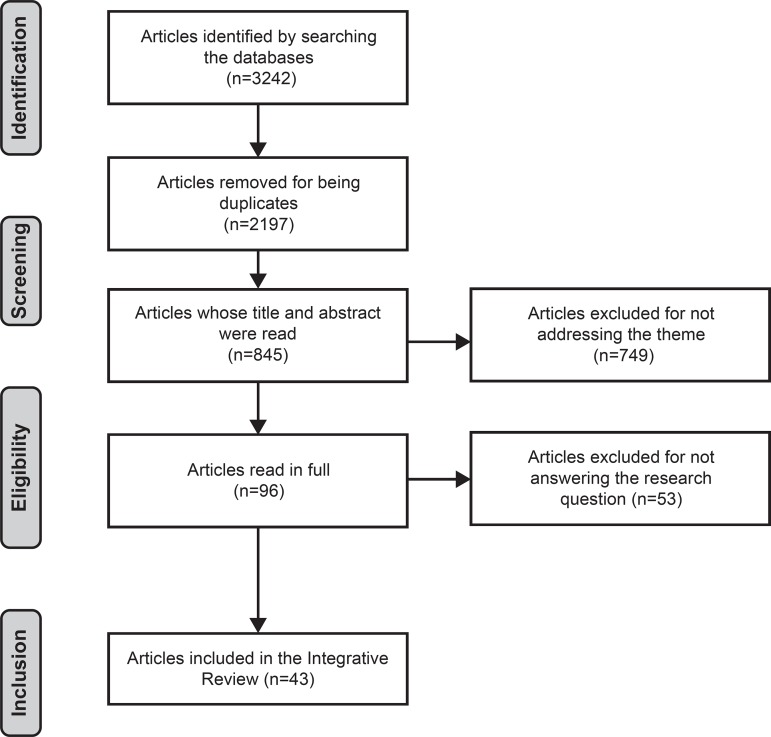
Informative flowchart of the integrative review’s phases, Campinas, SP, Brazil, 2017

The search in the databases reached a total of 3242 publications and, of these, 43 had content deemed relevant for the construction of the conceptual and operational definitions of the NO under study (Figure 1).

The same terms and keywords described above were used in the bibliographic search in the gray literature, and the following were found: one thesis^(^ 19 ^)^ in the Diva database; one dissertation^(^ 20 ^)^ in the South African National ETD Portal; one dissertation^(^ 21 ^)^ and one thesis^(^ 22 ^)^ in the Capes Theses and Dissertations Catalog; and one dissertation^(^ 23 ^)^ and three theses^(^ 24 ^-^ 26 ^)^ in ProQuest. In addition, three books^(^ 27 ^-^ 29 ^)^ and two manuals of the Ministry of Health^(^ 2 ^.^ 30 ^)^ were used.

The selected articles were carefully read and screened separately by two researchers, and the differences between the results were resolved by consensus with the presence of a third researcher. In this process, a validated instrument was used to categorize the data with the following elements: identification of the journal, institution hosting the study, methodological characteristics of the study, and assessment of methodological rigor^(^ 31 ^)^. Afterwards, an information sheet was built with data from the publications selected, including: authors, title, journal, country, language, year of publication, objective, methodological design, population, results and evidence level. The studies’ evidence level was classified according to Melnyk and Fineout-Overholt^(^ 32 ^)^.

## Results

To construct the operational and conceptual definitions of the indicators of the two NO^(^ 9 ^)^, 56 items were used, 43 of them articles, five theses, three dissertations, three books and two manuals.

Most of the productions were published in major Nursing or Medical journals. The prevailing language was English (n=45), followed by Portuguese (n=9) and Spanish (n=2).

In relation to methodological design, three were cohort studies^(^ 33 ^-^ 35 ^)^, four were longitudinal studies^(^ 36 ^-^ 39 ^)^, six were cross-sectional studies^(^ 20 ^,^ 24 ^,^ 26 ^,^ 40 ^-^ 42 ^)^, nine were descriptive studies^(^ 23 ^,^ 27 ^,^ 43 ^-^ 50 ^)^, and 29 were qualitative studies^(^ 19 ^,^ 21 ^,^ 51 ^-^ 75 ^)^. The evidence level that stood out was IV^(^ 32 ^)^, and the studies were conducted in Maternities (n=15), Outpatient Clinics (n=11), Neonatal Intensive Care Units (n=10), Breastfeeding Support Groups (n=6), Basic Health Units (n=5), and the participants’ home (n=4).

The conceptual and operational definitions of the indicators of NO “Breastfeeding establishment: maternal (1001)” and “Breastfeeding establishment: infant (1000)” are presented next^(^ 9 ^)^.

NO “Breastfeeding establishment: maternal” (1001)^(^ 9 ^)^ is defined as the “Maternal establishment of proper attachment of an infant to and sucking from the breast for nourishment during the first 3 weeks of breastfeeding”. The 18 indicators of this NO are presented below, as well as their respective conceptual and operational definitions.

*Comfort of position during nursing (100101)* - Conceptual definition: The mother feels comfortable when breastfeeding, regardless of the chosen position. Operational definition: The mother feels comfortable in the position she has chosen; thus, position is not a factor interfering with the pleasure felt and the time spent breastfeeding. Regardless of the position adopted by the mother, she maintains it with a calm face, relaxed shoulders and neck, and fully supported body and limbs^(^ 30 ^,^ 36 ^,^ 54 ^-^ 55 ^,^ 59 ^-^ 60 ^,^ 71 ^)^.
*Supports breast using “C” hold (cupping)”(100102)* - Conceptual definition: The mother supports the breast by positioning her index, middle, ring and little fingers underneath it, and her thumb on top of it, forming the letter “C”; Operational definition: The mother supports the lower part of the breast with her index, middle, ring and little fingers, and positions her thumb on top of it, forming the letter “C”, without stretching or compressing the skin, as well as without preventing the baby from approaching and latching onto it. She simulates a scissor with her hand, supporting the breast between the middle and index fingers^(^ 27 ^-^ 30 ^,^ 39 ^,^ 55 ^,^ 60 ^)^.
*Breast fullness prior to feeding (100103)* - Conceptual definition: The breast contains milk before the baby is positioned to start sucking. Operational definition: Upon inspection or palpation, observe whether the breasts contain milk before the baby is positioned to start sucking^(^ 27 ^,^ 45 ^-^ 46 ^,^ 53 ^,^ 55 ^,^ 57 ^,^ 59 ^,^ 74 ^)^.
*Milk ejection (let-down) reflex (100104)* - Conceptual definition: A reflex in which the milk contained in the breast is ejected out of it through the mammary ducts, as a result of the action of oxytocin, which causes the myoepithelial cells of the mammary gland’s smooth muscle to contract, upon stimulus triggered by the baby. Operational definition: Observe the spontaneous ejection of milk through one or both nipples, when the mother smells or thinks of the baby, or when the baby cries or is breastfed and milk leaks from the contralateral breast. The woman may describe small shocks or a tingling or pricking sensation in the breasts, before and during breastfeeding^(^ 21 ^,^ 28 ^-^ 29 ^,^ 33 ^,^ 49 ^,^ 60 ^,^ 63 ^,^ 65 ^)^.
*Recognition of infant swallowing (100106)* - Conceptual definition: Recognition by the mother and/or examiner of the baby’s effective swallowing when breastfed. Operational definition: When observing the baby in the breast, perceiving or hearing regular and smooth swallowing, in response to the presence of milk in the oropharynx^(^ 38 ^-^ 39 ^,^ 44 ^-^ 45 ^,^ 52 ^,^ 61 ^,^ 65 ^,^ 68 ^,^ 70 ^-^ 71 ^)^.
*Suction broken before removing infant from breast (100107)* - Conceptual definition: The mother removes the baby from the breast properly, so as not to cause pain or discomfort in the nipple. Operational definition: Observe if, when interrupting the breastfeeding process, the mother places her index or little finger in the baby’s mouth through the lip commissure, removing the nipple, so that the suction is interrupted before the child is removed from the breast^(^ 28 ^,^ 30 ^)^.
*Techniques to prevent nipple tenderness (100121)* - Conceptual definition: The mother is knowledgeable about techniques that help prevent nipple sensitivity. Operational definition: Observe, when talking to the mother, if she is knowledgeable about techniques that help prevent nipple sensitivity^(^ 21 ^,^ 36 ^,^ 44 ^,^ 46 ^,^ 53 ^,^ 55 ^,^ 59 ^,^ 63 ^,^ 70 ^,^ 73 ^-^ 75 ^).^
*Avoidance of artificial nipple use with infant (100109)* - Conceptual definition: The mother does not offer artificial nipples, pacifiers or bottles to the baby. Operational definition: Observe, when talking to the mother, if she offers only the maternal breast to feed her child, who she nurtures without using artificial nipples, holding the infant on her lap or offering the breast instead^(^ 2 ^,^ 30 ^,^ 35 ^,^ 39 ^,^ 44 ^,^ 67 ^-^ 68 ^,^ 73 ^)^.
*Avoidance of giving water to infant* (100110) - Conceptual definition: The mother breastfeeds exclusively, on demand, until the baby’s sixth month of life. Operational definition: Verify if the mother reports not offering water or other liquids to the baby, breastfeeding exclusively, on demand, in the first six months of life^(^ 29 ^,^ 49 ^,^ 52 ^-^ 54 ^,^ 59 ^-^ 60 ^,^ 65 ^-^ 66 ^)^.
*Supplemental feedings (100122)* - Conceptual definition: The mother offers complementary foods to the baby only when prescribed by a healthcare professional. Operational definition: Verify if the mother reports offering complementary foods only when prescribed by a health professional, prioritizing breast milk^(^ 29 ^,^ 49 ^,^ 52 ^-^ 54 ^,^ 59 ^-^ 60 ^,^ 65 ^-^ 66 ^)^.
*Recognition of early hunger signs (100113)* - Conceptual definition: The mother recognizes the first signs the baby shows when hungry. Operational definition: Verify if the mother recognizes or reports the hunger signs shown by the baby: becoming alert; putting hands and fingers inside mouth, making suckling movements; sticking tongue out; showing a search reflex; becoming irritated (kicking or squirming) and crying^(^ 20 ^,^ 35 ^,^ 38 ^,^ 44 ^,^ 52 ^,^ 61 ^,^ 65 ^,^ 68 ^,^ 71 ^)^.
*Response to the infant’s temperament (100112)* - Conceptual definition: The mother promptly responds to the infant’s temperament. Operational definition: Verify if the mother readily identifies the signs of temperament, such as crying, pained expressions and primitive reflexes^(^ 20 ^,^ 35 ^,^ 38 ^,^ 44 ^,^ 52 ^,^ 61 ^,^ 65 ^,^ 68 ^,^ 71 ^)^.
*Fluid intake of mother (100120)* - Conceptual definition: Adequate intake of fluids by the mother during breastfeeding. Operational definition: verify if the mother ingests at least two liters of fluids daily during the period when the baby is being breastfed^(^ 44 ^,^ 52 ^,^ 64 ^-^ 65 ^,^ 68 ^,^ 71 ^)^.
*Pumping of breast (100123)* - Conceptual definition: Removal of milk from the breast, manually or using a breast pump, to provide relief from discomfort when the breast is full or turgid, to increase milk production, or to store it. Operational definition: Verify if the mother is able to perform and/or describe the extraction of milk, either manually or using a breast pump, reporting its usefulness and frequency, as well as the appropriate technique, which involves hygiene measures along with proper positioning and use of the pump, to provide relief from discomfort, to increase milk production, or to store it^(^ 25 ^,^ 29 ^,^ 44 ^,^ 51 ^,^ 55 ^,^ 57 ^,^ 59 ^,^ 72 ^)^.
*Safe storage of breastmilk (100115)* - Conceptual definition: Storage of breast milk, after extracting it manually or using a breast pump, in containers and place that ensure the maintenance of the milk’s quality, free from contamination by microorganisms or other contaminants. Operational definition: verify if the mother reports taking all the necessary precautions for the safe storage of milk, or observe her taking them^(^ 25 ^,^ 29 ^-^ 30 ^,^ 44 ^,^ 51 ^,^ 55 ^-^ 56 ^,^ 59 ^,^ 72 ^)^.
*Use of family support (100124)* - Conceptual definition: The mother identifies sources of support for breastfeeding in her family and relies on them. Operational definition: observe or verify if the mother reports receiving encouragement and support from her family, who provides assistance by helping with household chores, caring for other children, and/or with breastfeeding itself^(^ 24 ^,^ 37 ^,^ 40 ^-^ 41 ^,^ 44 ^-^ 45 ^,^ 53 ^-^ 54 ^,^ 57 ^-^ 59 ^,^ 66 ^-^ 69 ^,^ 72 ^)^.
*Use of community support (100125)* - Conceptual definition: The mother identifies sources of support for breastfeeding in the community and relies on them. Operational definition: Observe or verify if the mother reports receiving encouragement and support from the community through support groups and networks^(^ 19 ^,^ 23 ^,^ 26 ^,^ 41 ^,^ 67 ^,^ 72 ^)^.
*Satisfaction with breastfeeding process (100118)* - Conceptual definition: Extension of the mother’s positive perception of the breastfeeding process. Operational definition: Verify if the mother declares feeling satisfied when breastfeeding her baby, and also in relation to the Breastfeeding establishment process^(^ 24 ^,^ 37 ^,^ 40 ^-^ 41 ^,^ 44 ^-^ 45 ^,^ 53 ^,^ 57 ^-^ 59 ^,^ 61 ^-^ 62 ^,^ 66 ^-^ 67 ^,^ 69 ^,^ 72 ^).^

NO “Breastfeeding establishment: infant” is defined as “Infant attachment to and sucking from the mother’s breast nourishment during the 3 first weeks of breastfeeding”^(^ 9 ^)^. The 12 indicators of this NO are presented below, as well as their respective conceptual and operational definitions.

*Proper alignment and latch on (100001)* - Conceptual definition: Adequate alignment of the baby in relation to the breast, favoring his/her latch and the extraction of milk. Operational definition: Observe if the baby’s ear, shoulder and hip are aligned, so that the neck is not turned or bent forward or backward. The baby’s body should be kept close to the mother’s, completely facing her, and his/her buttocks should be supported to keep the head at the breast’s level, with the mouth facing the nipple and the chin touching the breast^(^ 25 ^,^ 29 ^,^ 36 ^,^ 69 ^-^ 70 ^,^ 74 ^-^ 75 ^)^. These definitions do not cover the latch, as it was best described in the next indicator.
*Proper areolar grasp (100002)* - Conceptual definition: Attachment of the baby’s mouth to the breast, with lips wrapped around the nipple along with a large part of the areola, forming a perfect seal between the baby’s and the mother’s skin, ensuring the creation of a vacuum. Operational definition: Observe if the baby’s mouth is open wide, lips turned outwards, wrapped around the nipple and a large part of the areola (2 to 3 centimeters), with a greater portion of the areola visible above the mouth than below it, and chin touching the breast^(^ 2 ^,^ 27 ^-^ 30 ^,^ 47 ^-^ 48 ^,^ 50 ^)^.
*Proper areolar compression (100003)* - Conceptual definition: Compression exerted by the mouth of the newborn or infant on the nipple-areola complex, being one of the mechanisms that result in the extraction of milk from the breast while maintaining the integrity of the skin. Operational definition: Verify the mother’s report on the intensity of the compression exerted by the newborn/infant’s mouth on the nipple-areola complex, extracting milk without causing discomfort/pain, deformity or injury^(^ 2 ^,^ 27 ^-^ 30 ^,^ 47 ^-^ 48 ^,^ 50 ^)^.
*Correct tongue placement (100013)* - Conceptual definition: Tongue placement that favors milk extraction. Operational definition: Observe if, when sucking the maternal nipple/aeolar region, the baby’s tongue protrudes forward, supported on the lower gum but extending over it, with elevation of its lateral edges and formation of a groove in the central region (cannulation), gently involving the nipple and part of the areola, while performing rhythmic undulatory movements from the tip to the back. The tongue should be in an anteriorized and lowered position, extending over the lower gum and involving the nipple-areola complex, which the professional can evaluate by observing the anteriorization of the tongue upon insertion of a gloved finger in the mouth^(^ 2 ^,^ 27 ^-^ 30 ^,^ 47 ^-^ 48 ^,^ 50 ^)^.
*Suck reflex* (100014) - Conceptual definition: Primitive reflex responsible for the extraction of milk from the maternal breast, consisting of rhythmic undulatory movements of the tongue and jaw. Operational definition: Observe if the baby’s lips are attached to the maternal breast, and if his/her tongue makes rhythmic undulatory movements from the tip to the back, while the jaw moves downwards, when the mouth is fully open, then upwards to gently compress the nipple-areola complex, and backwards to follow the tongue in this process. During suction, there should be no snapping noises, and the cheeks should remain round while the tongue and jaw are moving^(^ 27 ^,^ 29 ^,^ 48 ^,^ 50 ^,^ 64 ^)^.
*Audible swallowing (100005)* - Conceptual definition: The baby’s swallowing while sucking on the maternal breast is audible, due to the presence of milk in the posterior pharynx. Operational definition: After a few rounds of deep and adequate suction, the baby’s swallowing can be heard with or without a stethoscope, due to the presence of milk in the posterior pharynx^(^ 27 ^,^ 29 ^,^ 48 ^,^ 50 ^,^ 64 ^)^.
*Nursing a minimum of 5 to 10 minutes per breast (100006)* - Conceptual definition: The baby keeps sucking on each breast for a minimum of 5 to 10 minutes. Operational definition: Observe if the baby remains on each breast, with good latch and effective suction, for a minimum of 5 to 10 minutes, without interruption^(^ 27 ^-^ 30 ^,^ 38 ^)^.
*Minimum of 8 feedings per day (100007)* - Conceptual definition: The baby is breastfed at least eight times per day. Operational definition: Verify if the mother/nursing team changes the baby’s diaper at least six times a day, observing clear urine^(^ 27 ^-^ 28 ^,^ 35 ^,^ 38 ^,^ 52 ^,^ 72 ^,^ 75 ^)^.
*Urinations per day appropriate for age (100008)* - Conceptual definition: Urinary elimination at least six times per day, with excretion of clear and diluted urine. Operational definition: Verify if the mother/nursing team changes the baby’s diaper at least six times a day, observing clear urine^(^ 27 ^-^ 28 ^,^ 30 ^,^ 34 ^,^ 44 ^)^.
*Loose, yellow, seedy stools per day appropriate for age (100009)* - Conceptual definition: Elimination of stools characteristic of breastfeeding babies, according to age. Operational definition: The mother/nursing team observes, in the first three days of life, the elimination of dark viscous-looking stools; between the 4th and 7th days of life, of greenish and loose stools; and from the 8th day of life on, of loose, yellow and granular stools. The mother/nursing team observes the presence of stools in 24 hours or in up to 5-7 days, without abdominal distention^(^ 27 ^-^ 28 ^,^ 30 ^,^ 34 ^,^ 44 ^)^.
*Weight gain appropriate for age (100010)* - Conceptual definition: Weight gain ranging from 15 to 25g per day for breastfeeding babies. Operational definition: The professional weighs the baby unclothed and without a diaper, observing a weight gain of 15 to 25g per day^(^ 2 ^,^ 27 ^,^ 30 ^,^ 35 ^,^ 38 ^,^ 52 ^,^ 64 ^,^ 75 ^)^.
*Infant contentment after feeding (100011)* - Conceptual definition: Presence of signs demonstrating that the newborn/infant is satisfied after breastfeeding. Operational definition: The mother/nursing team observes that the baby’s sucking slows down and he/she lets go of the nipple-areola region spontaneously, showing no search reflex when stimulated. The baby becomes relaxed and may start napping after breastfeeding^(^ 25 ^,^ 29 ^,^ 49 ^,^ 70 ^,^ 75 ^)^.

## Discussion

The breastfeeding establishment is a complex process that involves biological, social, cultural and emotional factors, both for the woman and her child. The evaluation and support of nurses and their team during breastfeeding are important to reduce early weaning, especially in premature babies. From the identification of barriers, such as latch and positioning difficulties, breast engorgement and nipple fissure, nurses are able to propose desirable results for the Breastfeeding establishment to happen^(^ 7 ^,^ 76 ^)^. Thus, the use of NO related to this process can help them identify states that interfere with the initial interaction and the adjustment of responses between mother and baby.

Regarding the title of outcome “Breastfeeding establishment: infant,” depending on language and reference, “infant” may not be synonymous with “newborn”. In English, infant is understood as a breastfeeding child, which could be a newborn, but also a baby who is between one and 23 months old. On the other hand, newborn is the term used to describe children with up to 28 days of life, a specific definition for the neonatal period. Considering that the Breastfeeding establishment can occur both in the neonatal period and after it, and that each period has its own specific aspects, changing the NO’s title to “Breastfeeding establishment: newborn & infant” is suggested. This proposal is made to increase the accuracy of the terms used in the title of this NO, according to definitions proposed by WHO^(^ 77 ^-^ 78 ^)^, as well as to highlight the specificity of each period. In addition, this suggestion aims to follow the standard of the NOC’s other outcomes, such as “Risk & security control”; “Disposal: liquids & electrolytes”.

Thus, there is also need to review the definitions of the two NO studied, as their content focuses on “the first three weeks of breastfeeding.” From the IR, it was found that the definition of these NO should not have this time limitation, since no scientific support for this period was identified, especially considering the individual responses of mother and child, particularly in cases of prematurity and hospitalization. In this way, defining NO “Breastfeeding establishment: newborn & infant (1000)” as “Newborn or infant’s latch and suction of the maternal nipple-areola complex for nutrition during the first weeks of breastfeeding” is suggested. As for NO “Breastfeeding establishment: maternal(1001),” it is suggested that its definition is changed to “adequate maternal establishment of latch and suction of the nipple-areola complex for the nutrition of newborns or infants during the first weeks of breastfeeding.”

Regarding the indicators of NO “Breastfeeding establishment: maternal(1001),” it is suggested that seven of them are reviewed, which will be specified below.

“Full breast before breastfeeding (100103)” could be changed to “Presence of colostrum/milk in the breast before breastfeeding,” since “full breast” is subject to variations in interpretation. In addition, considering the period when the evaluation is performed, there may be presence of colostrum or milk; thus, this change would make the indicator more specific^(^ 19 ^-^ 20 ^,^ 53 ^,^ 57 ^)^.

“Avoids giving water to the infant (100110)” could be changed to “Avoids offering water and other liquids to the newborn or infant,” since not only water, but other liquids, such as teas and juices, can also interfere with the Breastfeeding establishment^(^ 52 ^-^ 53 ^,^ 65 ^)^.

Indicator “Supplementary feeding (100122)” could be changed to “Complementary feeding, when indicated,” to avoid the existing confusion between the terms “supplementary” and “complementary.” In the context of breastfeeding, complementary foods are those offered to the child in concomitance with breast milk, when the latter is not sufficient for nutritional support and other foods or liquids are needed^(^ 28 ^,^ 79 ^)^, which occurs after six months of age or earlier, in case of weaning. On the other hand, supplementary foods are those containing specific nutrients that could not have been obtained by ingesting other foods^(^ 80 ^)^.

As for indicator “Response to infant’s temperament (100112),” it is not considered to be directly related to this NO, so its exclusion is recommended. Indicator “Recognition of early hunger signs (100113)” better contemplates the state that is intended to be described in the context of Breastfeeding establishment.

“Breast pumping (100123)” is clear and objective in its English version, but the translation into Brazilian Portuguese could be changed to “*Extração de colostro ou leite da mama*” (Extraction of colostrum or breast milk) to better represent the practice implied, which can be performed manually or using a breast pump.

From the IR, it was found that indicator “Use of community support (100125)” would better reflect the current context if it were changed to “Use of support from the community, social media and health services,” since women now use different support resources, such as breastfeeding groups, consultants and social media, as well as public health services^(^ 45 ^,^ 55 ^,^ 59 ^,^ 62 ^)^.

Changes in three indicators of NO “Breastfeeding establishment: infant” (1000) are suggested. The first one is “Proper alignment and latch (100001),” considering that this indicator could be divided into two: “Proper alignment of the newborn/infant in relation to the breast,” and “Proper areolar latch.” This proposal is due to the fact that they can be evaluated independently. In addition, it is considered that the indicator describing alignment should be included in NO “Breastfeeding establishment: maternal(1001),” because the baby’s alignment depends more on the mother than on the baby him/herself^(^ 27 ^-^ 29 ^)^. Indicator “Audible swallowing (100005)” could be changed to “Audible or noticeable swallowing,” since swallowing can be both heard and perceived visually^(^ 29 ^,^ 48 ^,^ 50 ^)^.

The authors of the present study suggest the removal of indicator “Interrupts breastfeeding at frequent intervals to position the infant for burping (100015)”, since it is not a fundamental event for the Breastfeeding establishment, especially as an ability of the baby. Thus, its conceptual and operational definitions were not developed, especially because interrupting breastfeeding “at frequent intervals” to perform this maneuver is not supported in the literature as a desirable indicator in this context.

The evidence level of the studies identified is considered a limitation of this review. None of them had evidence level I or II, although the contents presented were extremely important for the construction of the definitions. Another limitation was the absence of NO validation studies related to breastfeeding, which indicates there is an urgent need for further research to assess the application of the NOC.

## Conclusion

The present study allowed developing the conceptual and operational definitions of the indicators of NO “Breastfeeding establishment: infant (1000)” and “Breastfeeding establishment: maternal (1001),” with changes in the descriptions of both and in the title of the former. For the first one, changes to seven indicators and exclusion of one were suggested, while for the second, it was suggested to change two indicators and exclude one. The analysis of the NO in question, in contrast to the literature consulted, made it possible to identify the need for adjustments in the Portuguese version.

The improvement of the NOC as a standardized nursing language requires that the components of the NO clearly describe what nurses may find in their clinical practice, in addition to offering support for the development of research and teaching. Thus, the construction of conceptual and operational definitions is a contribution that helps this improvement process, and also supports the evaluation of the effectiveness of interventions, when monitoring changes in the patient’s state. In addition, the NO must be subjected to a semantic analysis by a committee of specialists made up of nurses with clinical experience and scientific knowledge, to refine the findings of the literature review.

There is an urgent need for studies validating NO related to breastfeeding in different contexts, as they will help refine the indicators used by nurses for the clinical evaluation of babies and their mothers. The NOC is considered to have great potential to assist in the qualification of the assessment records pertaining to the breastfeeding process, being also a tool for use in teaching and research.
